# Prenatal and perinatal risk factors for disability in a rural Nepali birth cohort

**DOI:** 10.1136/bmjgh-2017-000312

**Published:** 2017-08-06

**Authors:** Edward J N Haworth, Kirti M Tumbahangphe, Anthony Costello, Dharma Manandhar, Dhruba Adhikari, Bharat Budhathoki, Dej Krishna Shrestha, Khadka Sagar, Michelle Heys

**Affiliations:** 1 Great Ormond Street UCL Institute for Child Health, University College London, London, UK; 2 Mother and Infant Research Activities, Kathmandu, Nepal; 3 Department of Maternal, Newborn, Child and Adolescent Health (MCA), World Health Organization, Geneva, Switzerland; 4 UCL Institute for Global Health, University College London, London, UK

**Keywords:** child health, Paediatrics, cohort study, epidemiology, health policy

## Abstract

**Background:**

Improving newborn health remains a global health priority. Little however is known about the neurodevelopmental consequences for survivors of complications in pregnancy, labour and the neonatal period in in low-income countries outside of small selective and typically urban facility studies. We ask which antenatal, birth and neonatal factors are associated with disability in childhood in a large community birth cohort from rural Nepal.

**Methods:**

6436 infants were recruited during a cluster randomised control trial (RCT) of participatory women's groups (ISRCTN31137309), of whom 6075 survived beyond 28 days. At mean age of 11∙5 years (range 9.5–13.1), 4219 children (27% lost to follow-up) were available for disability screening which was conducted by face-to-face interview using the Module on Child Functioning and Disability produced by the Washington Group/UNICEF. Hypothesised risk factors for disability underwent multivariable regression modelling.

**Findings:**

Overall prevalence of disability was 7.4%. Maternal underweight (OR 1.44 (95% CI 1.01–2.08)), maternal cohabitation under 16 years of age (OR 1.50 (1.13–2.00)), standardised infant weight at 1 month (OR 0.82 (0.71–0.95)) and reported infant diarrhoea and vomiting in the first month (OR 2.48 (1.58–3.89)) were significantly associated with disability adjusted for trial allocation. The majority of hypothesised risk factors, including prematurity, were not significant.

**Interpretation:**

Proxies for early marriage and low birth weight and a measure of maternal undernutrition were associated with increased odds of disability. The lack of association of most other recognised risk factors for adverse outcome and disability may be due to survival bias.

**Key questions**

**What is already known about this topic?**

- In 2010, the global burden of disease survey estimated that 10% of disability adjusted life years were related to neonatal causes.
- In low-income countries, most of this burden is presumed to be from premature mortality.
- A 2013 review by Blencowe and colleagues highlighted a substantial gap in the evidence from low-income countries with most follow-up data based on small, high-risk cohorts.

**What are the new findings?**

- This is the first large, community recruited birth cohort from a low-income country to examine the relationship between known prenatal and perinatal risk factors with a positive disability screening tool at mean age 11.5 years.
- We found that the majority of known risk factors in this cohort, including prematurity, were not associated with later disability.
- Proxies for early marriage and low birth weight and a measure of maternal undernutrition were associated with increased odds of disability.

**Recommendations for policy**

- Our findings broadly agree with the consensus that in high mortality low-income settings survival bias likely means most known perinatal risk factors are not associated with later disability.
- This relationship will need to be reassessed as neonatal mortality rates continue to fall in countries like Nepal.
- Our findings also emphasise the relationship between early marriage, poverty, malnutrition and poor developmental outcomes and the need to break this cycle.

## Background

The Millennium Development Goals led to a renewed focus on reducing neonatal mortality in low-income country (LIC) settings but there has been a lack of attention to morbidity in survivors.^1^ The Sustainable Development Goals place an increased emphasis on disability, especially information gathering and inclusivity.^2 3^ The Global Burden of Disease Survey in 2010 estimated that nearly 10% of global disability-adjusted life years were due to neonatal conditions with the large majority of this due to early death.^4^ In high and middle-income countries, increasing survival of premature, small and sick neonates has led to increases in the number of disabled survivors.^5^ In contrast, there is little evidence in LIC exploring the impact of problems during pregnancy, birth and the neonatal period on disability in surviving infants and children. A 2013 review by Blencowe and colleagues highlighted ‘substantial challenges in terms of limitations in the current data’, and these gaps were worse in LIC with most data based on the follow-up of small, selective and typically urban facility-based studies.^6^ In particular, there is a dearth of large, long-term, population-based studies on the consequences of complications in pregnancy, labour and the neonatal period such as birth asphyxia, low birth weight (LBW), prematurity and neonatal infection.^7 8^ Rather, the majority of such studies are from middle-income countries, small (<300 participants) and/or of high-risk infants (either preterm or LBW) recruited from high-level facilities with a length of follow-up typically 2 years or less.^9–11^

Sixty-one per cent of the world's children live in low and lower middle income countries, defined by The World Bank in 2015 as being those with a Gross National Income (GNI) per capita of US$4035 or less 12. There are 31 LICs, with a GNI of US$1025 or less, including Nepal which is one of just 3 LIC outside of sub-Saharan Africa.^12^ The aim of this study was to explore prenatal and perinatal risk factors for childhood disability in a rural, community-based study of 4119 Nepali children at mean age of 11.5 years, thus addressing a major gap in the current literature around prevalence and risk factors for disability in this understudied setting.

## Methods

### Study setting

Nepal has a growing population of about 28.5 million people and a gross per capita income of US $730.^9^ Makwanpur District is a rural district in the central region of Nepal covering 19 000 km^2^ of mixed mountainous terrain, with a population of more than 500 000 people. It has one district hospital with a ratio of 7852 people per hospital bed. In 2001–2003 when the study was recruited, the neonatal mortality rate was 39/1000 live births, 94% of babies in rural areas were born at home and just 13% with a skilled birth attendant.^5^ Most households in Makwanpur District are dependent on subsistence agriculture.

### Background to original trial

This is a longitudinal study of a cohort of 6436 maternal infant dyads recruited to a cluster Randomised Control Trial (RCT) of perinatal women's participatory groups in Makwanpur, Nepal, which showed a sustainable and low cost decrease in neonatal and maternal mortality.^5^ Detailed methodology of the original study is reported elsewhere.^5^ The study clusters were village development committees, local government divisions, of which there are 43 in Makwanpur District. Clusters were paired on basis of similar topography, ethnicity and population density. One cluster was excluded due to security concerns and 12 of the remaining 21 pairs were selected by random to the study. Each cluster had an average population of 7000 and area of 60 km^2^. Clusters were mapped on foot between 1999 and 2000 to identify every household, followed by a baseline census to identify all female residents. In 2001, every eligible woman (married and aged 15–49) was interviewed and consented into the study. Recruitment took place between October 2001 and November 2003. Participants were visited monthly, and a pregnancy was registered when a woman ceased menstruation for 3 months in the absence of other explanation. Participants were interviewed 4 weeks postpartum for main outcomes of neonatal and maternal mortality. Thus, the baseline cohort was an entirely representative sample. In 2014, a long-term follow-up study of surviving and consenting participants was conducted and baseline (postpartum interview) and long-term follow-up data paired.

### Long-term follow-up study

Two rounds of follow-up interviews took place across the study site, 24 pairs of interviewers visited families and if the family had moved attempts to trace them via neighbours were made. Seven supervisors directly observed 18% of all interviews. Data described here were collected during the first round of follow-up interviews and included anthropometry, forward and backwards digit recall testing, and a disability screening questionnaire.^10^ Interviewers were high school graduates and received a week's training in use of the screening tool.

Ethical approval for the study was obtained both from Nepal Health Research Council and the University College London research ethics committee. Informed verbal consent was granted by all participants prior to participation, which is more culturally appropriate in this setting than written consent.

### Outcome

The main outcome was disability as measured at face-to-face interview using the Module on Child Functioning and Disability (MCFD).^10^ The MCFD is a new tool developed by the Washington Group on Disability Statistics and UNICEF, to improve and standardise information gathering on childhood disability. It is designed for use in children and young people aged 2–17 years, building on the Short Set of Questions that has been validated for use in adults and previously used in Nepal.^11^ We used it with permission from the Washington Group during the final stages of validation testing. It is a questionnaire taken by the child's main caregiver to assess functioning across a number of domains: speech and language, hearing, vision, cognition, mobility, self-care and emotions and behaviour. Owing to the current absence of validity data on the questions around emotions and behaviour, the UNICEF and the Washington Group have advised the definition of disability to be restricted to the core functional domains of speech and language, hearing, vision, learning, mobility and motor skills (MCFD core). Responses are usually ranked: *no difficulty (1), some difficulty (2), a lot of difficulty (3)*, *cannot do at all (4)*. We defined a positive disability screen as at least some difficulty (>=2), in at least one domain. This is to use the most inclusive definition of disability and because of the very low prevalence of other definitions in this study (supplementary appendix table A1)

10.1136/bmjgh-2017-000312.supp1 Supplementary file 1

### Prenatal and perinatal risk factors

To minimise the pitfalls of post hoc testing, prior to reviewing the dataset a list of factors known to be associated with poor developmental outcomes was drawn up. Maternal characteristics, antenatal care and complications, problems during labour, essential newborn care, harmful traditional practices and neonatal illness were included.^13 14^ The dataset was then reviewed to see whether information on these factors was collected or if there were biologically plausible proxies available. Potential prenatal and perinatal risk factors were categorised into four groups: maternal, antenatal, labour and neonatal. Table 1 summarises the potential data available for each category and the final variables selected.

**Table 1:** Variable selection

Original hypothesised variable	Information available	Final variable
Maternal		
Maternal age	Age	Maternal age
Parity	-	-
Age at first pregnancy	Age of marriage, age of cohabitation with husband	Cohabitation<16 years old
Literacy and education	Literacy, years of school completed.	Illiterate
Nutritional status	Height, weight, MUAC, months of household food insecurity	Underweight (BMI<18∙5) Chronic (>2 months) food insecurity
Antenatal		
Antenatal care	Any antenatal care, four skilled visits	Any antenatal care four skilled antenatal visits
Preeclampsia	Headache, blurred vision, swollen face, short of breath, high blood pressure if checked	Symptoms of preeclampsia (any positive)
Eclampsia	Seizure in late pregnancy, seizure in labour	Presumed eclampsia (either positive)
Gestational diabetes	-	-
Labour and delivery		
Place of birth	Place of birth	Facility birth (any hospital or clinic)
Skilled birth attendant	Who attended birth (alone, family, neighbour, community health worker, health assistant, midwife, nurse, doctor)	Skilled birth attendant (doctor, nurse, midwife, health assistant)
Prolonged labour	Labour duration	Prolonged labour (>20 hours)
Presenting part	Presenting part	Presenting part not head
Type of delivery	Type of delivery	Type of delivery (vaginal, instrumental, caesarean)
Induction and monitoring	-	-
Multiple birth	Twin pregnancy Birth order	Twin pregnancy
Risk of infection	Fever in labour, prolonged rupture of membranes, foul swelling discharge, dysuria in late pregnancy	Any risk of infection
Antepartum haemorrhage and placental abruption	Antepartum haemorrhage	Antepartum haemorrhage
Hygiene	Attendant washed hands, use of clean delivery kit	Kit use Hand washing
Foetal distress	Meconium	Meconium
Birth asphyxia	-	-
Neonatal		
Prematurity	GA (months) by LMP	Preterm (GA<9 months)
Gender	Gender	Gender
Birth weight	Subjective size at birth (‘very small’, ‘smaller than usual’, ‘normal’), weight at 1 month	Very small Standardised weight at 1 month
Thermal care	Heated delivery room	Heating
Delayed bathing	Time to first bath	First bath at<6 hours old
Clean cord care	Type of blade used to cut cord, application of substances to cord	Sterile blade Dry cord care
Early initiation of breast feeding	Time to initiate breast feeding	Initiated within first hour
Discarding colostrum	Discarded colostrum	Discarded colostrum
Exclusive breast feeding	Other milk given, solids given, water given	Any supplemental feeds (first month)
Neonatal jaundice	-	-
Neonatal infection	Fever, cough, fast breathing, recession, diarrhoea and vomiting, dysentery	Any infant illness Fever Respiratory distress (fast breathing or recession) Diarrhoea and vomiting

BMI, body mass index; GA, gestational age; LMP, last monthly period; MUAC, mid upper arm circumference.

Of note, as 93% of infants were classified as ‘smaller than usual’ at birth, we chose ‘very small’ as the best marker for LBW. Gestational age (GA) was calculated based on last monthly period (LMP) as recalled by the mother, it is only available as completed month's gestation and 96% of infants were born with a reported GA of 9 months. For epidemiological-based studies in these types of settings, this is the only method available with which to calculate GA. Antenatal ultrasound was, and by and large is still, not available in this and similar settings and as the majority of babies were born at home, clinical gestational assessment at birth was also not an option. Very few infants were born at <8 months (0.7%) and for analysis all infants <9 months (<36 weeks) are classified as preterm. Skilled birth attendance is defined by WHO criteria and includes ‘mid-level’ workers such as health assistants but excludes community health workers who may nonetheless have undergone some obstetric training.^15^

### Potential confounders

Potential confounders considered were trial allocation and baseline socioeconomic status (SES) as measured by household asset ownership score. Asset-based scores have been shown to be a reliable and simple way to assess differences in SES in LIC.^16 17^ Asset ownership was measured on a scale of 0–3, with1 indicating no assets on list, 1: basic assets such as clock or radio, 2: more expensive assets such as sewing machine or hand tractor and 3: very expensive assets such as a motor vehicle.

### Anthropometric measures

Weight was measured to the closest 100 g using a Tanita HS302 Solar Scale with participants in minimal clothing, stood in the centre of the scale with arms at sides and face forward. Standing heights of participants were measured to the nearest mm against a Shorr board. Both height and weight are the average of two measurements unless they differed by more than 4 mm or 400 g, respectively, in which case a third was taken and the average of the two closest was used. Stunting was defined as a height for age z-score <−2 according to WHO 2006 standards. Weight at 1 month was standardised to create a mean of 0 and SD of 1.

### Statistical analysis

Univariate analysis was performed by Pearson χ^2^ for categorical variables, by two sample t-test for normally distributed continuous variables, and Mann-Whitney U test for non-normally distributed continuous variables. Collinearity was assessed by variance inflation factor scores with a score >10 indicating potentially significant collinearity. For some of the delivery variables such as hand washing, there were some ‘don't knows’ which are treated as missing data.

To build the final model, all the variables were ranked by their significance on univariate analysis and variables with a significance of <0.1 included in initial model. A backwards stepwise regression modelling approach was taken after included variables were checked for collinearity. Starting with the least significant variable on univariate analysis, variables were dropped and the model compared with the previous model by likelihood ratio testing. Variables were dropped permanently if the significance of the likelihood ratio test was ≥0.1 and retained if <0.1. The model was checked for confounding by trial allocation and SES. A variable was considered significantly confounded if the adjusted OR changed by more than 10% from the unadjusted model. The final models were model 1 (unadjusted), model 2 (adjusted by original trial allocation) and model 3 (adjusted by household asset score). Due to missing data, only 3318 of the 4419 children screened for disability were included in the regression model which was carried out as a complete case analysis.

To calculate population attributable risk (PAR), the relative risk (RR) was first estimated using log binomial regression. PAR (%) was then calculated using the formula [Pe(RR−1)]/[1+Pe(RR−10], where Pe is the prevalence of the exposure in the population that was screened for disability.

Statistical analysis was performed using Stata V. 13.^18^

### Role of the funding source

The design of this study and all data collection, analyses, interpretation and the writing of the report were performed without the sponsors' involvement. Full access to data was granted to the corresponding author. All authors participated in the study design or analysis, and approved the submission of the manuscript.

## Results

**Figure 1:**
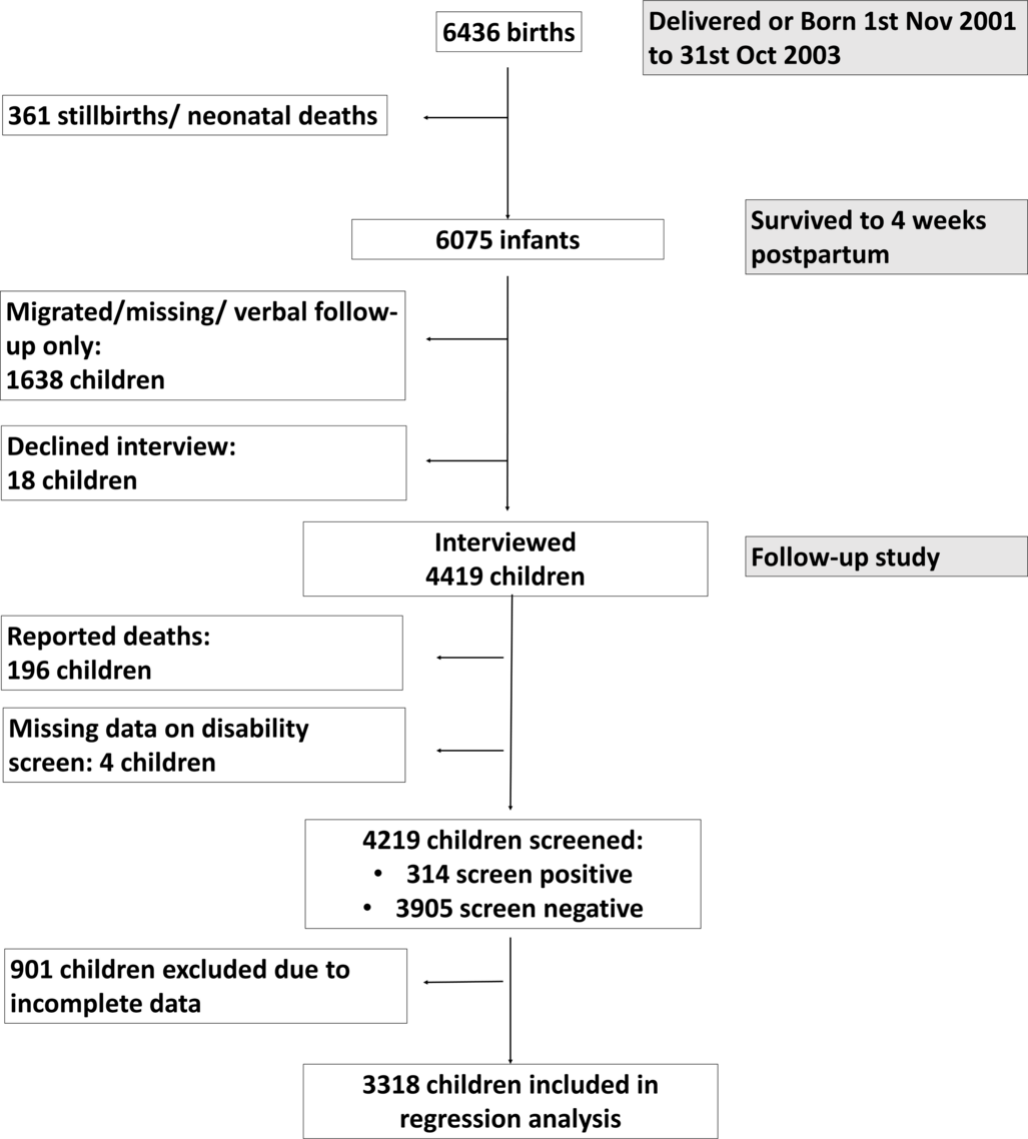
Flowchart of study participants.

Figure 1 shows the flow diagram of study participants from baseline trial to long-term follow-up.

The mean age of children for whom detailed follow-up was available (n=4419) was 11.5 years (range 9.5–13.1 years) and 51.5% were male. Fifty per cent were born to mothers in the intervention arm of the original trial. Table 2 shows the overall prevalence of a positive disability screen (7.4%) and details of disability prevalence by disability category. The prevalence of stunting was 39.4%.

**Table 2:** Prevalence of disability by type

At least some difficulty in at least one domain	314/4219 (7.4%)
A lot of difficulty in at least one domain	40/4219 (1.0%)
Cannot do at all in at least one domain	13/4219 (0.3%)
At least some difficulty in at least two domains	90/4219 (2.1%)

Table 3 shows a comparison of baseline characteristics of children whose families were interviewed (including those who died after 4 weeks of age) with those who were not interviewed (excluding those who died in the neonatal period).

**Table 3:** Baseline characteristics of children whose families were interviewed at long-term follow-up compared with those not interviewed. Including children who died after the original trial period (4 weeks postpartum) but excluding stillbirths and neonatal deaths.

	Interview	No interview	p Value
Allocation to intervention in original trial	2181/4414 (49%)	667/1607 (42%)	<0.001
Household appliance score: none of assets on list	2354/4413 (53%)	865/1606 (54%)	0.062
Household appliance score: clock, radio, iron, bike	1468/4413 (33%)	559/1606 (35%)	
Household appliance score: most expensive assets	252/4413 (6%)	64/1606 (4%)	
Median maternal age, years (IQR)	26.2 (22.7–31.8) n=4414	25.4 (22.4–30.9) n=1607	<0.001
Mother cohabited<16 years old	1048/4221 (25%)	391/1484 (26%)	0.246
Mother illiterate	2907/4221 (69%)	1018/1484 (69%)	0.846
Maternal underweight	489/4177 (12%)	178/1480 (12%)	0.743
Facility birth	173/4414 (4%)	71/1607 (4%)	0.385
Skilled birth attendant	111/4241 (3%)	52/1540 (3%)	0.123
Male gender	2276/4414 (52%)	855/1607 (53%)	0.259
Preterm (<36 weeks)	51/4414 (1%)	35/1607 (2%)	0.003
Infant size ‘very small’	130/4414 (3%)	57/1607 (4%)	0.234
Supplemental feeds in neonatal period	260/4406 (6%)	132/1603 (8%)	0.001
Mean weight in grams at 1 month (SD)	4264 (908) n=3825	4220 (914) n=1349	0.124
Any illness in neonatal period	1611/4414 (37%)	639/1607 (40%)	0.020

Table 4 describes the relationship between the independent variables and the primary outcome of positive disability screening. Highlighted variables had a univariate significance of p<0.1 and were included in the initial regression model.

**Table 4:** Description of variables and relationship to disability screening

Highlighted variables had a univariant significance p<0.1 and were included in initial model			p Value
	Negative disability screen	Positive disability screen	
Maternal			
Median maternal age, years (IQR)	26.2 (22.7–31.6) n=3905	26.4 (22.6–32.1) n=314	0.509
Mother cohabited<16 years old	**894/3730 (24%)**	**96/304 (32%)**	**0.003**
Mother illiterate	2549/3730 (68%)	213/304 (70%)	0.533
Underweight (body mass index<18.5)	**424/3694 (11%)**	**47/299 (16%)**	**0.029**
Chronic food insecurity	2214/3904 (57%)	174/314 (55%)	0.655
Antenatal			
Any antenatal care	1733/3905 (44%)	144/314 (46%)	0.611
Four skilled antenatal care visits	**363/3902 (9%)**	**20/314 (6%)**	**0.082**
Symptoms of preeclampsia	**2015/3775 (53%)**	**181/308 (59%)**	**0.068**
Presumed eclampsia	732/3903 (19%)	68/314 (22%)	0.207
Labour and delivery			
Delivered in healthcare facility	162/3905 (4%)	8/314 (3%)	0.165
Skilled birth attendant	**106/3747 (3%)**	**3/304 (1%)**	**0.056**
Prolonged labour (>20 hours)	1124/3905 (29%)	99/314 (32%)	0.302
Presenting part not head	18/3875 (<1%)	1/312 (<1%)	0.716
Any assisted delivery	59/3905 (2%)	6/314 (2%)	0.580
Multiple birth	54/3905 (1%)	4/314 (1%)	0.873
Any risk of infection	1890/3408 (55%)	164/273 (60%)	0.140
Antepartum haemorrhage	526/3903 (13%)	38/314 (12%)	0.491
Clean delivery kit used	457/3905 (12%)	32/314 (10%)	0.421
Attendant washed hands	1925/2669 (72%)	150/221 (68%)	0.177
Meconium	615/2315 (27%)	53/195 (27%)	0.852
Neonatal			
Preterm (<36 weeks)	40/3905 (1%)	4/314 (1%)	0.675
Male gender	2000/3905 (51%)	171/314 (54%)	0.269
Size ‘very small’	**102/3905 (3%)**	**18/314 (6%)**	**<0.001**
Delivery room heated	**3071/3905 (79%)**	**261/314 (83%)**	**0.061**
Mean weight in grams at 1 month (SD)	**4299 (902) n=3389**	**4066 (906) n=264**	**0.001**
First bath<6 hours old	568/3901 (15%)	42/314 (13%)	0.566
Substance applied to cord	741/3820 (19%)	70/308 (23%)	0.147
Sterile blade cut cord	1608/3869 (42%)	137/313 (44%)	0.446
Discarded colostrum	1346/3898 (35%)	118/314 (38%)	0.257
Breast feeding started<1 hour old	2341/3898 (60%)	175/314 (56%)	0.133
Any supplemental feeds	220/3898 (6%)	22/314 (7%)	0.318
Any illness	1388/3905 (36%)	130/314 (41%)	0.037
Respiratory distress	421/3905 (11%)	38/314 (12%)	0.470
Diarrhoea and persistent vomiting	**172/3905 (4%)**	**33/314 (11%)**	**<0.001**
Fever	**586/3905 (15%)**	**59/314 (19%)**	**0.073**
Potential confounders			
Allocation to intervention in original trial	1969/3905 (50%)	133/314 (42%)	0.006
Household appliance score: none of assets on list	2071/3904 (50%)	158/314 (53%)	0.351
Household appliance score: most expensive assets	230/3904 (6%)	15/314 (5%)	0.417

**Figure 2:**
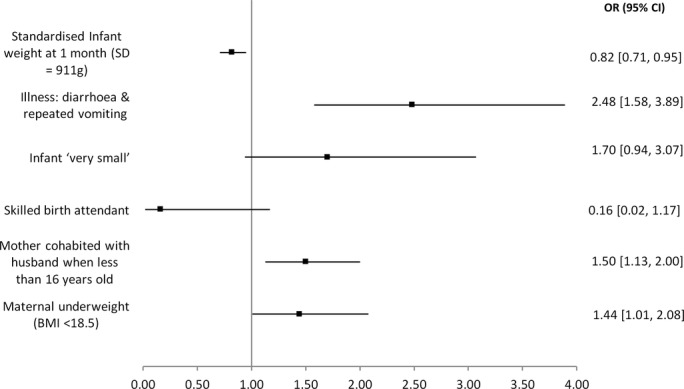
Unadjusted ORs for disability. n=3318. BMI, body mass index.

Table 5 and figure 2 show the final logistic regression model for disability. Maternal underweight (OR 1∙44 (95% CI 1∙01–2∙08)), maternal cohabitation under 16 years of age (OR 1∙50 (1∙13–2∙00)), standardised weight at 1 month (OR 0∙82 (0∙71–0∙95)) and reported diarrhoea and vomiting (OR 2∙48 (1∙58–3∙89)) were significantly associated with screening positive for disability. The majority of hypothesised risk factors were not statistically significant on multivariable analysis. Table 5 also shows the population attributable risk, estimated RR, used to calculate it was very similar to the OR and is not shown.

**Table 5:** Final logistic regression model for disability (3318 children included) and population attributable risk

	Model 1	Model 2	Model 3	Population attributable risk (95% CI)
	Unadjusted OR (95% CI)	OR adjusted by trial allocation	OR adjusted by Household Appliance Score	
Prenatal				
Maternal underweight (BMI<18.5)	1.44 (1.01 to 2.08)	1.45 (1.01 to 2.09)	1.44 (1.00 to 2.08)	4.6% (0.2% to 5.7%)
Mother cohabited with husband when <16 years old	1.50 (1.13 to 2.00)	1.50 (1.12 to 1.99)	1.52 (1.14 to 2.02)	10.3% (3.0% to 11.6%)
Labour and delivery				
Skilled birth attendant	0.16 (0.02 to 1.17)	0.17 (0.02 to 1.22)	0.15 (0.02 to 1.12)	−2.2% (−2.7% to 5.9%)
Neonatal				
Infant ‘very small’	1.70 (0.94 to 3.07)	1.75 (0.96 to 3.16)	1.66 (0.92 to 3.01)	1.7% (−0.1% to 4.5%)
Illness: diarrhoea and repeated vomiting	2.48 (1.58 to 3.89)	2.45 (1.57,3.85)	2.48 (1.58 to 3.88)	5.7% (2.6% to 9.9%)
Standardised Infant weight at 1 month	0.82 (0.71 to 0.95)	0.82 (0.71 to 0.95)	0.81 (0.70 to 0.94)	

BMI, body mass index.

Table 6 compares the characteristics of children who are included in the regression model and those who were screened for disability but not included in the model due to missing data.

**Table 6:** Characteristics of those included in and those dropped from regression modelling due to data completeness.

	Disability screening but not in regression model	In regression model	p Value	Number of missing values/4219 (%)
Allocation to intervention in original trial	316/901 (35%)	1786/3318 (54%)	<0.001	
Household appliance score: none of assets on list	475/901 (53%)	1754/3318 (53%)	0.801	
Household appliance score: most expensive assets	58/901 (6%)	187/3318 (6%)		
Positive disability screen	73/901 (8%)	241/3318 (7%)	0.395	
Included in initial regression model but dropped from final model				
Four skilled antenatal care visits	87/901 (10%)	296/3315 (9%)	0.501	3 (<1%)
Possible symptoms of preeclampsia	499/873 (57%)	1697/3210 (53%)	0.024	136 (3%)
Delivery room heated	681/901 (76%)	2651/3318 (80%)	0.005	0
Infant had fever in neonatal period	156/901 (17%)	489/3318 (15%)	0.057	0
Retained in final logistic regression model				
Maternal underweight (BMI<18∙5)	89/675 (13%)	382/3318 (12%)	0.220	226 (5%)
Mother cohabited with husband when <16 years old	178/716 (25%)	812/3318 (24%)	0.827	185 (4%)
Skilled birth attendant	18/733 (3%)	91/3318 (3%)	0.664	168 (4%)
Infant size ‘very small’	12/901 (1%)	108/3318 (3%)	0.002	0
Infant illness: diarrhoea and repeated vomiting	46/901 (5%)	159/3318 (5%)	0.698	0
Mean weight in grams at 1 month (SD)	4395(963) n=335	4271(898) n=3318	0.017	566 (13%)

BMI, body mass index.

## Discussion

We suspect that survival bias is likely behind the lack of association between many known risk factors and later disability in this high burden cohort. Two hundred and eighty-eight (4.5%) of the total births were ‘very small’ but 110 of these were either stillborn or known to have died leaving just 2.9% of those screened. Likewise, of the 197 infants born preterm 118 (60%) had died before follow-up.

Increased weight at 1 month was negatively associated with disability. Weight at 1 month is clearly linked to birth weight but will also be significantly affected by the neonatal period and is therefore not directly comparable. Maternal underweight is a known risk factor for LBW and preterm delivery and in severe cases may affect factors such as breast milk quality and supply leading to health consequences for infants.^19 20^ Additionally, maternal micronutrient deficiency such as iron or iodine will have knock-on effects for the fetus and nursing infant. Iron deficiency anaemia is recognised as one the leading causes of child disability and cognitive impairment.^21^

Age of maternal cohabitation with husband is used here as a proxy for age at first pregnancy and early cohabitation is positively associated with childhood disability. Adolescent pregnancy is known to cause cessation of linear growth, and this is especially relevant in a population with high rates of childhood stunting who often undergo prolonged periods of catch-up growth.^22^ This can lead to increased risk of cephalopelvic disproportion and obstructed labour in future pregnancies.^23^ Both malnutrition and early marriage are associated with lower SES however, and these associations may be partly explained by residual confounding for poverty.^24^

A history of any neonatal illness, breathing difficulties or neonatal fever was not significantly associated with disability. It is possible again that survival bias is important here, perhaps with diarrhoea and vomiting being relatively mild symptoms and more severely ill infants being unlikely to survive. For instance, of the 865 infants who developed breathing problems in the neonatal period, 176 (20%) had died prior to follow-up.

The presence of a skilled birth attendant also approached a significant association with decreased odds of disability. In a population like Makwanpur, skilled birth attendance is heavily confounded as it is much more likely when there are complications of labour.^25^ In a post hoc analysis (table 7), we examined the association of skilled birth attendance with high-risk deliveries and found that women who had a skilled birth attendant present were more likely to have reported antenatal haemorrhage and prolonged labour. Despite this confounding, the evidence from this cohort suggests that the impact of skilled care at delivery may extend far beyond birth.

**Table 7:** Skilled birth attendance rate and labour complications

Skilled birth attendance rate		p Value
No antepartum haemorrhage: 161/5136 (3.1%)	Antepartum haemorrhage: 41/828 (5.0%)	0.01
No prolonged labour: 100/4140 (2.4%)	Prolonged labour: 102/1835 (5.6%)	<0.001

### Limitations to original trial data

The limitations to the original trial were several fold, for logistic reasons, collecting birth weight was not feasible and complications of labour and pregnancy were reported symptomatically around a month after the event, some degree of recall bias is likely. The nearest proxy to birth weight is a subjective categorisation of size at birth but only 4.5% of children were born ‘very small’ and this measure likely significantly underestimates the prevalence of LBW in this cohort compared with other studies.^24 25^ The overall rate of preterm birth in this cohort was just 3%, which is much lower than the 16% reported by Wu and the 21% by Christian in Nepal.^26 27^ This is unlikely to be due solely to the misclassification of infants at 36–37 weeks of gestation as term and likely reflects the uncertainties of using recalled LMP for GA assessment in this setting. Infants who are classed as ‘very small’ will include growth-restricted term infants and those born preterm, both growth restricted and appropriately sized. There is unfortunately no way to disaggregate these groups adequately in this study; however, only 30% of ‘very small’ infants were also classed as preterm.

### Bias

The current study design is retrospective and opportunistic, and this has inherent limitations. We have aimed to minimise the risks of ‘data dredging’ by using a preconceived hypothesised list of variables, but there was a process of compromise involved in consummating this list with the available data. The total loss to follow-up was 27.3% which is excellent for a study of this size and setting. Most of those lost had moved out of the area, and there was no practical way to assess them without using substantial extra resources. As seen in table 3, there are some differences between those followed up and those lost. Most differences are small and of limited relevance even where statistically significant. There is however quite a marked difference between the two groups in original trial allocation which may suggest some interview bias, perhaps with more effort being made to obtain interviews in intervention clusters. This is seen again in table 6, as those who children from intervention clusters are less likely to have been dropped from the model due to missing data perhaps reflecting a greater effort to collect baseline data. Missing data meant 21.4% of those screened for disability could not be included in our model. There is no significant difference in the rates of positive disability screen between those included and those not. We have treated this missing data as being missing completely at random. However, there are some differences between the groups, particularly in infant size, and these may impact on our model.

### Module on child functioning and disability

Since this study was undertaken, the results of validation testing of the MCFD in children from India and Cameron have been published and taken with the prevalence found here suggest that cultural interpretation of degree of difficulty may influence reporting and/or that differences in administration may lead to variation in interpretation of scales of difficulty.^28^ Using our definition of *at least some difficulty*, in at least one domain they found a prevalence of 64% in Cameroon and 35% in India compared with 7.4% here. Using the stricter definition of *at least a lot of difficulty*, they found a prevalence of 9% in Cameroon and 4% in India to our 1%. However, of note our prevalence of 7.4% is in keeping with a systematic review of the global estimates of childhood disability in low and middle-income countries showing that despite a wide range in estimates (0.5%–18%), the majority clustered around 5%–10%.^29^ This suggests that our participants and interviewers could differentiate between scales of difficulty but more work on cultural interpretation of degrees of difficulty is required. Further studies of the MCFD would also be helpful to explore these issues, including formal neurodevelopmental assessment of children who screen positive for disability.

### Strengths and importance

Despite these limitations, this is a unique and important study that examines the relationship between antenatal, birth and neonatal risk factors and later disability in a large birth cohort in a high burden country. A wealth of data collected during the original trial allowed a detailed assessment of birth environment. The cohort was recruited in the community where most infants are born, in contrast to the majority of small and facility recruited cohorts. The length of follow-up allows a more accurate assessment of later childhood disability as shorter follow-up periods can underestimate prevalence. This study addresses this previously discussed gap and for a community recruited cohort in an LIC it is relatively large. A review of the literature found it is twice the size of the nearest comparable study, whose primary outcome was cognitive test scores rather than disability screening.^26^

### Policy implications and next steps

Child marriage is recognised as both a symptom and cause of poverty. In Nepal, girls married under 20 are 20% less likely to have access to education and girls in the highest wealth quintile marry on average 2 years later than those in the lowest.^30^ Given the limitations of this study, caution must be used when extrapolating to policy. However, as the population attributable risk in table 5 shows, action in this area could have great potential impact and further research is warranted. Likewise, the link between maternal undernutrition, LBW and neonatal morbidity, childhood stunting and poverty and ill health in later life is well recognised and supported by this study.^31^ Tackling this remains a great challenge and requires integrated strategies to support female education, secure livelihoods and food security, sanitation and clean drinking water.^32^

Our main finding that most risk factors are not associated with later disability is on the surface surprising, and we speculate that this is likely to be due to high mortality and survival bias in this population. Further data are required to confirm or refute this hypothesis and it is to be hoped that other large birth cohorts recruited from perinatal intervention studies in LIC, such as those from other perinatal women's group trials, will also be followed up to later childhood.

Since this cohort was born, the neonatal mortality rate in Nepal has declined from 39 to 23 per 1000 live births.^33^ With declining neonatal mortality rates, it will be important to revisit the link between the birth environment and disability in countries like Nepal as decreasing mortality could be expected to lead to greater morbidity in later childhood.

## Conclusions

In this birth cohort from rural Nepal, the lack of association between most perinatal risk factors and later childhood disability may be due to high mortality and survival bias. However, proxies for early marriage and LBW and a measure of maternal undernutrition were associated with increased odds of disability. The relationship between poverty, maternal malnutrition and poor neurodevelopmental outcome is well recognised, and our findings here emphasise the importance of breaking this cycle.

## References

[1] Lawn JE, Blencowe H, Oza S, et al Every Newborn: progress, priorities, and potential beyond survival. Lancet 2014;384:189–205. 10.1016/S0140-6736(14)60496-7 24853593

[2] United Nations Division for Social Policy and Development. Disability Inclusive Sustainable Development Goals. 2015 http://www.un.org/disabilities/documents/sdgs/disability_inclusive_sdgs.pdf (accessed 01/08/2016).

[3] Tardi R, Njelesani J Disability and the post-2015 development agenda. Disabil Rehabil 2015;37:1496–500. 10.3109/09638288.2014.972589 25350660

[4] Murray CJ, Vos T, Lozano R, et al Disability-adjusted life years (DALYs) for 291 diseases and injuries in 21 regions, 1990-2010: a systematic analysis for the global burden of disease study 2010. Lancet 2012;380:2197–223. 10.1016/S0140-6736(12)61689-4 23245608

[5] Manandhar DS, Osrin D, Shrestha BP, et al Members of the MIRA Makwanpur trial team. Effect of a participatory intervention with women's groups on birth outcomes in Nepal: cluster-randomised controlled trial. Lancet 2004;364:970–9. 10.1016/S0140-6736(04)17021-9 15364188

[6] Blencowe H, Vos T, Lee AC, et al Estimates of neonatal morbidities and disabilities at regional and global levels for 2010: introduction, methods overview, and relevant findings from the global burden of disease study. Pediatr Res 2013;74(Suppl 1):4–16. 10.1038/pr.2013.203 24366460 PMC3873708

[7] Lee AC, Kozuki N, Blencowe H, et al Intrapartum-related neonatal encephalopathy incidence and impairment at regional and global levels for 2010 with trends from 1990. Pediatr Res 2013;74 Suppl 1(Suppl 1):50–72. 10.1038/pr.2013.206 24366463 PMC3873711

[8] Seale AC, Blencowe H, Zaidi A, et al Neonatal Infections Estimation Team. Neonatal severe bacterial infection impairment estimates in South Asia, sub-Saharan Africa, and Latin America for 2010. Pediatr Res 2013;74(Suppl 1):73–85. 10.1038/pr.2013.207 24366464 PMC3873707

[9] Bank W, Nepal D South Asia. http://data.worldbank.org/?locations=NP-8S (Accessed 01/08/2016).

[10] Washington Group on Disability Statistics & UNICEF. Module on Child Functioning and Disability 2014 https://www.cdc.gov/nchs/data/washington_group/meeting13/wg13_unicef_child_disability_background.pdf (Accessed 01/08/2016).

[11] Washington Group on Disability Statistics. Overview of Implementation Protocols for Testing the Washington Group Short Set of Questions on Disability Rationale for and development of the WG questions. 2006 https://www.cdc.gov/nchs/data/washington_group/meeting6/main_implementation_protocol.pdf (Accessed 01/08/2016).

[12] Bank W Data for low income, lower middle income. http://data.worldbank.org/?locations=XM-XN (Accessed 01/05/2017).

[13] Mwaniki MK, Atieno M, Lawn JE, et al Long-term neurodevelopmental outcomes after intrauterine and neonatal insults: a systematic review. Lancet 2012;379:445–52. 10.1016/S0140-6736(11)61577-8 22244654 PMC3273721

[14] UNICEF. What Works for Children in South Asia. Newborn Care: An Overview 2004 https://www.unicef.org/rosa/Newborn.pdf (Accessed 01/08/2016).

[15] World Health Organisation. Making pregnancy safer: the critical role of the skilled attendant (. 2004 http://www.who.int/maternal_child_adolescent/documents/9241591692/en/ (Accessed 01/08/2016).

[16] Galobardes B, Lynch J, Smith GD Measuring socioeconomic position in health research. Br Med Bull 2007;81-82:21–37. 10.1093/bmb/ldm001 17284541

[17] Howe LD, Galobardes B, Matijasevich A, et al Measuring socio-economic position for epidemiological studies in low- and middle-income countries: a methods of measurement in epidemiology paper. Int J Epidemiol 2012;41:871–86. 10.1093/ije/dys037 22438428 PMC3396323

[18] StataCorp. Stata Statistical Software: release 13. College Station, TX: StataCorp, 2013.

[19] Sebire NJ, Jolly M, Harris J, et al Is maternal underweight really a risk factor for adverse pregnancy outcome? A population-based study in London. BJOG 2001;108:61–6. 11213006 10.1111/j.1471-0528.2001.00021.x

[20] Dewey KG Energy and protein requirements during lactation. Annu Rev Nutr 1997;17:19–36. 10.1146/annurev.nutr.17.1.19 9240917

[21] Stoltzfus RJ Iron-deficiency Anemia: reexamining the nature and magnitude of the public health problem. J. Nutr 2001;131:616–35. 10.1093/jn/131.2.697S 11160600

[22] Prentice AM, Ward KA, Goldberg GR, et al Critical windows for nutritional interventions against stunting. Am J Clin Nutr 2013;97:911–8. 10.3945/ajcn.112.052332 23553163 PMC3628381

[23] Konje JC, Ladipo OA Nutrition and obstructed labor. Am J Clin Nutr 2000;72:291S-297S. 10.1093/ajcn/72.1.291S 10871595

[24] Mitra S, Posarac A, Vick B Disability and poverty in developing countries: a multidimensional study. World Dev 2013;41:1–18. 10.1016/j.worlddev.2012.05.024

[25] Graham WJ, Bell JS, Bullough CH Can skilled attendance at delivery reduce maternal mortality in developing countries? *Safe Mother*. Strateg. a Rev. Evid 2001;17:97–130.

[26] Christian P, Murray-Kolb LE, Tielsch JM, et al Associations between preterm birth, small-for-gestational age, and neonatal morbidity and cognitive function among school-age children in Nepal. BMC Pediatr 2014;14:58 10.1186/1471-2431-14-58 24575933 PMC3974060

[27] Wu LA, Katz J, Mullany LC, et al The association of preterm birth and small birthweight for gestational age on childhood disability screening using the ten questions plus tool in rural Sarlahi district, southern Nepal. Child Care Health Dev 2012;38:332–40. 10.1111/j.1365-2214.2011.01221.x 21375569

[28] MacTaggart I, Cappa C, Kuper H Field testing a draft version of the UNICEF/Washington Group Module on child functioning and disability. Background, methodology and preliminary findings from Cameroon and India. Alter-European Journal of Disability Research 2016;0672. 10.1016/j.alter.2016.09.003 PMC6488935 31049115

[29] Maulik PK, Darmstadt GL Childhood disability in low- and middle-income countries: overview of screening, prevention, services, legislation, and epidemiology. Pediatrics 2007;120 Suppl 1:S1–S55. 10.1542/peds.2007-0043B 17603094

[30] UNICEF. Cost of Inaction, Child and Adolescent Marriage in Nepal, 2012 http://unicef.org.np/uploads/files/614784587893950542-final-working-paper-001.pdf (Accessed 01/08/2016).

[31] Prendergast AJ, Humphrey JH The stunting syndrome in developing countries. Paediatr Int Child Health 2014;34:250–65. 10.1179/2046905514Y.0000000158 25310000 PMC4232245

[32] Qar Z, et al Maternal and Child Undernutrition 3 what works? interventions for maternal and child undernutrition and survival. 371: The Lancet, 2008. 10.1016/S0140-6736(07)61693-6 18206226

[33] UNICEF. Child Mortality estimates Nepal. http://www.childmortality.org/index.php?r=site/graph#ID=NPL_Nepal (Accessed 01/08/2016).

