# Supplementary material for: Prenatal and perinatal risk factors for disability in a rural Nepali birth cohort
Source: BMJ Glob Health. 2017 Aug 6;2(3):e000312. doi: 10.1136/bmjgh-2017-000312 (PMC5656139; doi:10.1136/bmjgh-2017-000312)
Supplement: Supplementary file 1 [file bmjgh-2017-000312supp001.pdf]

Appendix

Table A1: Module on child functioning and disability			
Seeing	Children aged 2-17	Does [name] wear glasses or contact lenses?	1= Yes<br>0 =No
		[*if wears glasses*]<br>Does [name] have difficulty seeing, when wearing his/her glasses? Would you say…	1=No difficulty<br>2=Some difficulty<br>3=A lot of difficulty<br>4=Cannot do at all
		[*If child does NOT wear glasses*] Does [name] have difficulty seeing?	1=No difficulty<br>2=Some difficulty<br>3=A lot of difficulty<br>4=Cannot do at all
Hearing	Children aged 2-17	Does [name] use a hearing aid?	1= Yes<br>0 =No
		[*If child uses a hearing aid*] Does [name] have difficulty hearing, when using his/her hearing aid(s)?	1=No difficulty<br>2=Some difficulty<br>3=A lot of difficulty<br>4=Cannot do at all
		[*If child does NOT use a hearing aid*]<br>Does [name] have difficulty hearing?	1=No difficulty<br>2=Some difficulty<br>3=A lot of difficulty<br>4=Cannot do at all
Walking	Children aged 5-17	Compared with children of the same age, does [name] have difficulty walking 500 yards/meters on level ground? That would be about the length of 5 football fields.	1=No difficulty<br>2=Some difficulty<br>3=A lot of difficulty<br>4=Cannot do at all
		Compared with children of the same age, does [name] have difficulty walking 100 yards/meters on level ground? That would be about the length of 1 football field.	1=No difficulty<br>2=Some difficulty<br>3=A lot of difficulty<br>4=Cannot do at all
Self-care	Children aged 5-17	Compared with children of the same age, does [name] have difficulty with self-care such as feeding or dressing him/herself?	1=No difficulty<br>2=Some difficulty<br>3=A lot of difficulty<br>4=Cannot do at all
Communication and comprehension	Children aged 5-17	Compared with children of the same age and using [his/her] usual language, does [name] have difficulty understanding other people?	1=No difficulty<br>2=Some difficulty<br>3=A lot of difficulty<br>4=Cannot do at all
		Compared with children of the same age and using [his/her] usual language, does[name] have difficulty being understood by other people?	1=No difficulty<br>2=Some difficulty<br>3=A lot of difficulty<br>4=Cannot do at all
Learning	Children aged 3-17 years	Compared with children of the same age, does [name] have difficulty learning to do new things?	1=No difficulty<br>2=Some difficulty<br>3=A lot of difficulty<br>4=Cannot do at all
	Children aged 5-17	Compared with children of the same age, does [name] have difficulty remembering	1=No difficulty<br>2=Some difficulty

		things that they have learned?	3=A lot of difficulty<br>4=Cannot do at all
Emotions	Children aged 5-17	Compared with children of the same age, how much does [he /she] worry or feel sad? Would you say… [*Read response categories*]	1=The same or less<br>2=More<br>3=A lot more
Behaviour	Children aged 5-17	Compared with children of the same age, how much difficulty does [name] have controlling[his/her] behaviour?	1=No difficulty<br>2=Some difficulty<br>3=A lot of difficulty<br>4=Cannot do at all
Attention	Children aged 5-17	Compared with children of the same age, does [name] have difficulty completing a task?	1=No difficulty<br>2=Some difficulty<br>3=A lot of difficulty<br>4=Cannot do at all
Coping with change	Children aged 5-17	Compared with children of the same age, does [name] have difficulty accepting change to plans or routine?	1=No difficulty<br>2=Some difficulty<br>3=A lot of difficulty<br>4=Cannot do at all
Relationships	Children aged 5-17	Does [name] have difficulty getting along with children of [his/her] age?	1=No difficulty<br>2=Some difficulty<br>3=A lot of difficulty<br>4=Cannot do at all
Playing	Children aged 2-12 years	Compared with children of the same age, does [name] have difficulty playing with other children?	1=No difficulty<br>2=Some difficulty<br>3=A lot of difficulty<br>4=Cannot do at all
